# Whole Body Vibration Training on Muscle Strength and Brain-Derived Neurotrophic Factor Levels in Elderly Woman With Knee Osteoarthritis: A Randomized Clinical Trial Study

**DOI:** 10.3389/fphys.2019.00756

**Published:** 2019-06-25

**Authors:** Adriano Prado Simão, Vanessa Amaral Mendonça, Núbia Carelli Pereira Avelar, Sueli Ferreira da Fonseca, Jousielle Márcia Santos, Ana Carolina Coelho de Oliveira, Rosalina Tossige-Gomes, Vanessa Gonçalves César Ribeiro, Camila Danielle Cunha Neves, Cláudio Heitor Balthazar, Hércules Ribeiro Leite, Pedro Henrique Scheidt Figueiredo, Mário Bernardo-Filho, Ana Cristina Rodrigues Lacerda

**Affiliations:** ^1^ Departamento de Fisioterapia, Universidade Federal de Alfenas (UNIFAL), Alfenas, Brazil; ^2^ Centro Integrado de Pós-Graduação e Pesquisa em Saúde (CIPq-Saúde), Universidade Federal dos Vales do Jequitinhonha e Mucuri (UFVJM), Diamantina, Brazil; ^3^ Programa Multicêntrico de Pós-Graduação em Ciências Fisiológicas (PMPGCF), Sociedade Brasileira de Fisiologia (SBFis), Butanta, Brazil; ^4^ Programa de Pós-Graduação em Reabilitação e Desempenho Funcional (PPGReab), Universidade Federal dos Vales do Jequitinhonha e Mucuri (UFVJM), Diamantina, Brazil; ^5^ Departamento de Ciências da Saúde, Universidade Federal de Santa Catarina (UFSC), Araranguá, Brazil; ^6^ Departamento de Biofísica e Biometria, Instituto de Biologia Roberto Alcântara Gomes, Universidade Federal do Estado do Rio de Janeiro, Rio de Janeiro, Brazil

**Keywords:** muscle strength, squat, brain-derived neurotrophic factor, osteoarthritis of knee, whole body vibration

## Abstract

**Background**: Osteoarthritis of the knee (kOA) is a chronic, progressive, degenerative health condition that contributes to the imbalance between the synthesis and destruction of articular cartilage. Recently, whole body vibration (WBV) training has been recommended as an effective alternative for strength training in elderly people, and various physiological effects are obtained in response to exercise performed on a vibratory platform, such as an increase in muscle activation and improved muscle performance. However, the effects of WBV particularly on the strength of the quadriceps muscle and neuronal plasticity are unknown.

**Objective**: The aim of this study was to evaluate the effects of adding WBV to squat training on the isometric quadriceps muscle strength (IQMS) and the plasma levels of brain-derived neurotrophic factor (BDNF) in elderly woman with kOA.

**Methods**: Fifteen elderly women ≥65 years of age with kOA were randomized into two interventions: (1) the vibration group (VG), in which participants performed squat exercise training in association with WBV or (2) the exercise group (EG), in which participants performed squat exercise training without vibration, for 12 weeks 3×/week.

**Results**: Compared to the EG group, the VG group demonstrated a significantly greater delta (Δ) in IQMS values (IC95% 0.43–7.06; *p* ≤ 0.05) and in Δ BDNF plasma levels (IC95% −32.51 to 4.217; *p* ≤ 0.05) after the intervention period. There was an association between increase of Δ BDNF plasma levels and increase of Δ IQMS (*β* = 0.57; *R*^2^ = 0.32; *p* = 0.03).

**Conclusion**: The addition of WBV to squat exercise training improves lower limb muscle performance in elderly women with kOA. These findings suggest that the improvement in muscle performance is related to neuromuscular adaptations induced by WBV.

**Clinical Trial Registration**: www.ClinicalTrials.gov, identifier NCT03918291.

## Introduction

Osteoarthritis (OA) is a chronic, progressive, degenerative disease of multifactorial etiology involving biomechanical and genetic factors that contribute to the imbalance between the synthesis and destruction of articular cartilage (Goldring and Goldring, 2004) and characterized by arthralgia, stiffness, swelling, and decreased muscle strength and functionality (Zacaron et al., 2006; Michael et al., 2010). Osteoarthritis of the knee (kOA) is the most common form of OA, affecting approximately 7% of people aged 65–70 years and 11.2% of those aged over 80 years (Marx et al., 2006). The prevalence and incidence of OA differ by gender with females showing a significantly greater prevalence and severity of kOA (Srikanth et al., 2005).

The pain and the increase in intra-articular fluid, which are common in kOA, sensitize the capsular mechanoreceptors, which activate inhibitory interneurons in the spinal medulla and consequently decrease the muscle activation (e.g., the quadriceps). This phenomenon is called arthrogenic muscle inhibition and can lead to loss of muscle mass and impair performance of daily living activities such as walking, climbing stairs, and rising from a chair (McNair et al., 1996; Hautier and Bonnefoy, 2007). The reversal of this condition can be achieved by physical exercise. A study of our research group (Gomes et al., 2016) showed that the increased physical performance in elderly women with kOA may be related to the immunomodulatory effect of exercise. In addition, physical exercise induces a cascade of molecular and cellular processes that support brain plasticity. In this context, brain-derived neurotrophic factor (BDNF) appears to be the most susceptible to regulation induced by exercise and physical activity and could be linked to these mechanisms. The BDNF is a neurotrophin produced by several tissues, i.e., brain, muscle, and kidney, and has been involved with central and peripheral molecular processes of energy metabolism and homeostasis, development of immature neurons and survival of adult neurons, neuronal differentiation, and synaptic plasticity (Lindsay, 1994; Knaepen et al., 2010; Teixeira et al., 2010). Furthermore, it has been speculated about the role of BDNF as a myokine. Yu et al. (2017) demonstrated a time-dependent upregulation of BDNF in skeletal muscle of rats, which apparently is involved in the regeneration after exercise-induced muscle damage.

Recently, whole body vibration (WBV) training has been recommended as an effective alternative for strength training in elderly people (Roelants et al., 2004; Bogaerts et al., 2007), and various physiological effects are obtained in response to acute exercise performed on a vibratory platform, such as an increase in muscle activation (Abercromby et al., 2007), improved muscle performance (Cochrane et al., 2008, 2010). During this training modality, the individual stands on a platform that generates vertical sinusoidal vibrations. These mechanical stimuli are transmitted to the body where they stimulate the primary endings of the muscle spindles, which in turn activate α-motor neurons, resulting in muscle contractions known as tonic vibration reflex (Cardinale and Bosco, 2003). The WBV training is a safe, suitable, and effective training method and is a potentially feasible intervention for those patients who cannot participate in conventional strength training. In addition, WBV training could yield effects similar to regular strength training (Roelants et al., 2004; Trans et al., 2009), but with a lower load on the affected joint (Trans et al., 2009). Moreover, the time to perform the exercise is less, making it a faster form of training (Merriman and Jackson, 2009).

Studies demonstrated improvements on function, gait parameters, and quality of life in patients with kOA after a training program where WBV was added to squat exercise training (Wang et al., 2016a,b). Our research team also demonstrated improvements in the functionality and self-perception of disease status in elderly subjects with kOA after a 12-week training program in which WBV was added to squat exercise training (Avelar et al., 2011). However, to the best of our knowledge, only two studies have evaluated the effect of WBV training on muscle strength in elderly people with kOA (Trans et al., 2009; Bokaeian et al., 2016). In these studies, it was reported that the WBV-exercise regime on a platform increased muscle strength in subjects with kOA. Nevertheless, there is a gap in the literature regarding the possible mechanism related to the muscle performance improvement in this population. Studies suggest that adding WBV training to strengthening training may provide a better treatment effects for patients with kOA (Park et al., 2013; Bokaeian et al., 2016).

Because kOA in older adults contributes to a greater deterioration in muscle strength (Marx et al., 2006) and the reduction in muscle strength could be the result of changes in neuromuscular activation (McNair et al., 1996), it is believed that the addition of WBV to squat exercise training could promote an increase in muscle strength in older women with OA (Trans et al., 2009) Therefore, the present study was designed to investigate the effects of WBV in addition to squat exercise training in elderly women with kOA on the following parameters: (1) isometric strength of the quadriceps muscle (IQMS) and (2) BDNF plasma levels. Our hypothesis is that the addition of WBV to squat exercise training would increase the IQMS and the BDNF plasma levels in older women with kOA.

## Materials and Methods

### Ethical Statement

This study was carried out in accordance with the recommendations of the ethical principles for research involving humans (Resolution 196-96 of the National Health Council of the Brazilian Ministry of Health) and Federal University of Jequitinhonha and Mucuri Valleys Ethics Committee. All subjects gave written informed consent in accordance with the Declaration of Helsinki. The protocol was approved by the Federal University of Jequitinhonha and Mucuri Valleys Ethics Committee (protocol No. 046/08) and was registered in ClinicalTrials.gov (protocol ID: NCT03918291).

### Design

This was a randomized, controlled trial in which the variables were assessed 24 h before and after a 12-week training program. For the allocation of participants, a 1:1 ratio randomization was performed using opaque envelopes for allocation concealment. To minimize the chance of bias, we used the following methods: (1) opaque, sealed, and serial-numbered envelopes; (2) the envelopes were opened sequentially after the participant’s name and details were written on the envelope; and (3) the envelopes were kept in a locked and secure place. The allocation sequence was concealed from the researcher enrolling and assessing participants. Only one researcher performing the randomization was aware of the group assignment.

### Subjects

The volunteers were recruited from the physiotherapy clinic and medical referrals. To participate in the study, volunteers were required to meet the following inclusion criteria: females older than 65 years of age, not using hormone replacement therapy, and diagnosed with OA in at least one knee in accordance with of the American College of Rheumatology (Kellgren and Lawrence, 1957). The severity of the kOA was classified radiographically according to the Kellgren and Lawrence scale (grades 0–4, with 0 being normal and 4 representing severe OA). A Grade 2 classification (definite osteophytes and possible narrowing of joint space) was used as a cutoff to determine knee OA (Hinton et al., 2002; Schiphof et al., 2008). Exclusion criteria included the following: no recent knee injury, no walking aid requirement, and no rehabilitation procedures in the previous 3 months. Volunteers were also excluded if they had any orthopedic, neurological, respiratory, or acute cardiac diseases or if they had any cognitive deficit as determined by the Mini-Mental State Examination (Gomes et al., 2012).

**Figure 1:**
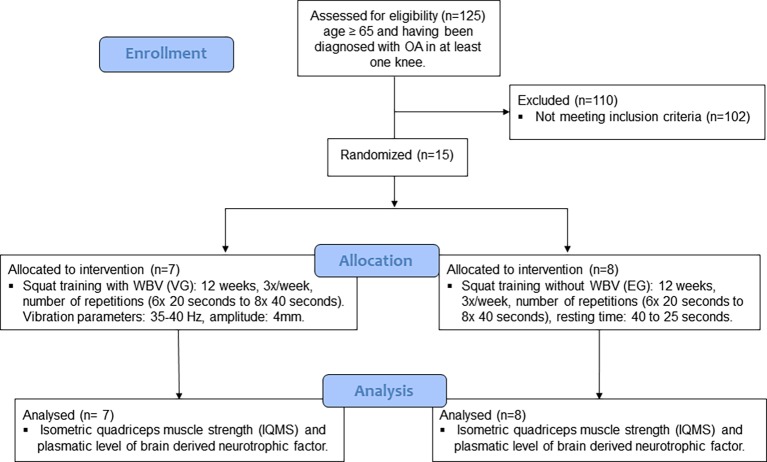
The study flow chart. OA, osteoarthritis; VG, vibration group; EG, exercise group; WBV, whole body vibration.

Of the 125 elderly people screened for eligibility, 15 elderly women fulfilled the criteria, had blood sampling collected at rest (8:00 a.m.), and then participated in the IQMS test. Thereafter, the subjects were allocated into two groups: (1) the vibration group, in which participants performed squat exercise training in association with WBV (VG; *n* = 7) or (2) the exercise group, in which participants performed squat exercise training without vibration (EG; *n* = 8; Figure 1).

### Procedures

The clinical and demographic data were collected from the participants using an evaluation chart. Prior to the initiation of the 12-week intervention program, a blood sample was collected at 8:00 a.m. from the volunteers of all groups followed by the IQMS evaluation. At 24 h after the intervention period, the volunteers of both groups were reassessed. These procedures were performed to avoid any circadian rhythm effects on hormonal status and the performance of IQMS. The tests were performed by an experienced examiner who was blind to the group allocation.

The intervention program consisted of performing squat exercise training with or without WBV, three times a week on alternate days.

#### Warm-Up

Prior to each training session, the participants warmed up on a stationary cycle (Stone Fitness, 2001, Huntertown, United States) at 70% of the predicted maximum heart rate for each subject’s age (220 – age) and were monitored using a Polar heart rate monitor (Polar F4, Kempele, Finland) for 10 min. Immediately afterwards, the participants of the VG group were placed in a position with their feet 28 cm apart (14 cm to the right and 14 cm to the left of the center of the vibration stimulus) to ensure that each of the lower limbs received the same amount of vibration stimulus and were ready to begin the squat exercises on the vibratory platform, while the participants of the EG group performed the same procedure without vibration.

#### Squat Exercises

The intensity of squat exercise training was systematically augmented in the VG and EG over the training period by increasing the number of repetitions (6 × 20 s to 8 × 40 s) and reducing the resting time (40–25 s). These parameters were based on the study by Avelar et al. The squat exercise was performed starting at approximately 10° of knee flexion and continuing until 60° of knee flexion was reached. The 60° angle was measured in each volunteer using a universal goniometer prior to initiating the exercise series, and a barrier was placed at the gluteal region to limit the degree of flexion of the knee. For temporal control during the squat, an examiner provided verbal encouragement to standardize the length of maintaining the semi full position (3 s) and the flexed position (3 s of isometric contraction) of the knees in each squat repetition. The participants of both groups were placed in position with their feet 28 cm apart.

#### Whole Body Vibration

For the volunteers in the VG group, a commercial model of a vibration platform was used (FitVibe, GymnaUniphy NV, Bilzen, Belgium). In this group, acceleration was also increased by varying the vibration frequency (35–40 Hz). The mechanical stimulation parameters of the vibration consisted of the following: frequency of 35–40 Hz, amplitude of 4 mm, and acceleration that ranged from 2.78 to 3.26 G. The platform provided a vertical sinusoidal vibration. Prior to initiating data collection, the platform acceleration values were verified using the Mega accelerometer (Acceleration Measuring Kit ZPP1-3D-147BC, Southampton, United Kingdom).

To measure acceleration on the horizontal and vertical axes, two accelerometers (Acceleration Measuring Kit ZPP1-3D-147BC, Southampton, United Kingdom) were fixed at a distance of 14 cm from the center of platform vibration. The signal was amplified electronically and was stored. This signal was obtained at a frequency of 1.000 Hz and was sent for computer analysis. Each accelerometer was calibrated using two calibration points, and applying zero and gravity, Earth’s gravity being 1 G (9.81 m/s^2^). To obtain the true acceleration values of the platform, the values of Earth’s gravity were subtracted along the vertical axis from the total signal received so that the acceleration of the platform would begin at 0 m/s^2^. The data were transferred to a computer using the Megawin software program, and the mean and maximum acceleration of each sample on each axis were analyzed using the Matlab software program. Each frequency used was measured over 60 s. In the pilot study, interexaminer reliability was found to be high, with a coefficient of variation of 1.05%.

The elderly underwent training on the vibratory platform with barefoot to avoid any damping effect due to different footwear (Marín et al., 2009). In addition, a predetermined distance from the feet (14 cm to the right and 14 cm to the left of the vibration center of the platform) was set to ensure that each of the lower limbs received the same amount of vibration stimulus. Moreover, with the aim of maintaining control of the body’s center of gravity behind the base of the support, the positioning of the spine, arms, and head and the type of squat (simulating the motion of sitting in a chair) were standardized.

#### Isometric Quadriceps Muscle Strength

The IQMS was evaluated by the maximal voluntary isometric contraction of knee extensors (MVIC) measured using the load cell according to the method described by Neves et al. (2011). For this evaluation, the volunteer was positioned in flexion-extension chair (Home Sport, Master Top model, Belo Horizonte, Brazil) and assumed a sitting posture with a straight trunk and the hips flexed at 90°. The limb to be tested was positioned at 60° of knee flexion (assessed individually by goniometry of the knee), and the resistance lever was positioned on the distal leg.

The load cell was properly calibrated and positioned near the leg extension perpendicular to the ground for signal acquisition during the isometric muscle strength tests (Miotec - Biomedical Equipment, Porto Alegre, Brazil). The load cell was connected to the software MIOTOOL 400 that transmitted the isometric muscle strength values to a computer. The MVIC test was performed for 6 s with 1 min between sets and with repeating three sets per leg.

Before the test, the volunteers warmed up for 10 min on a stationary bicycle (Stone Fitness, 2001, Huntertown, United State), which increased the heart rate (HR) to 70% of the estimated maximum HR by age, as measured with the use of the Polar heart monitors (Polar F4, Kempele, Finland). This procedure was performed to reduce the risk of injury during the test (Neves et al., 2011). The intra-class correlation coefficient (ICC) for IQMS was 0.98 (95% CI: 0.94–0.99).

#### Analysis of Brain-Derived Neurotrophic Factor by ELISA

For plasma processing, 10 ml peripheral blood samples were collected from the antecubital vein using aseptic techniques and heparin as an anticoagulant. The blood was immediately centrifuged twice at 3.000 *g* for 10 min, and the plasma was kept frozen at −70°C until assayed. Plasma BDNF levels were measured in duplicate using ELISA kits for BDNF (BDNF DuoSet, R&D Systems) according to the manufacturer’s instructions; the detection limit was 10 pg/ml.

### Statistical Analysis

The SPSS statistical software program (IBM, Chicago, IL, USA) was used for the statistical analyses. All data were expressed as means (confidence interval 95%). The Shapiro–Wilk test was used to evaluate the normality of the data. Because the dependent variables were normally distributed, parametric tests were used for the statistical analyses. An independent *t*-test was performed to assess the differences between the means. Thus, we used the delta analysis (pretest-posttest control group design) because allowed to verify the magnitude of the variation of the effect of the intervention, i.e., how much the addition of WBV contributed to squat training (VG) compared to squat training without WBV (EG) in the outcomes. The pretest-posttest control group design involves the random assignment of units to either a treatment or a control group. Successful randomization of units to groups in this experimental design, as well as similar between-groups anthropomorphic characteristics and outcomes at baseline (pretest), ensures that any pre-existing differences between the units in the treatment and control groups are due to chance and do not reflect systematic differences (Valente and MacKinnon, 2017). The correlation between the variables was evaluated using the Pearson coefficient. Simple linear regression models were performed to predict the BDNF and IQMS variables. Effect size (*d*) was checked in the G*Power program. Effect size conventions for test family (*t* tests) and two independent means: *d* = 0.20 (small), *d* = 0.50 (medium), *d* = 0.80 large. The effect size analysis is an additional measure to the traditional statistical test of the null hypothesis and aims to verify the clinical significance of the effect found and is not limited to dichotomous (significant or not significant) results. Thus, with the effect size analysis, it is possible to identify whether the observed differences are small, moderate, or large. The level of significance was set at *p* ≤ 0.05 for all the tests.

## Results

There were no significant differences between the groups regarding demographic and anthropometric data. No significant differences were found in the data collected prior to training between-groups, thus confirming the baseline homogeneity of the groups (Table 1).

**Table 1:** Sample characterization before training.

Characteristics	VG (*N* = 7)			EG (*N* = 8)			*p*
Age (years)	75 (68.5–81.5)			71 (67.7–74.3)			0.06
Body mass (kg)	72.75 (62.2–83.3)			74.24 (65.9–82.6)			0.75
Height (m)	1.58 (1.5–1.6)			1.57 (1.5–1.6)			0.81
Severity of knee OA^*^ (%)	2	3	4	2	3	4	–
	29	14	57	50	13	37	
IQMS before training (kg)	21.89 (19.5–24.3)			25.25 (21.8–28.7)			0.09
Plasma BDNF concentration before	4,778			3,043			0.06
training (pg/ml)	(2952.4–6603.7)			(1623.4–4462.6)			

* *Classified radiographically according to Kellgren and Lawrence scale*.

Data presented as means (confidence interval 95%). ^*^p ≤ 0.05.

*IQMS, isometric quadriceps muscle strength; BDNF, brain-derived neurotrophic factor; VG, vibration group; EG, exercise group*.

All participants in both intervention groups complied with the full intervention program. Overall compliance was 99.7% in the VG group and 98.6% in the EG group. There was no significant difference between the groups with respect to compliance (*p* = 0.41).

**Figure 2:**
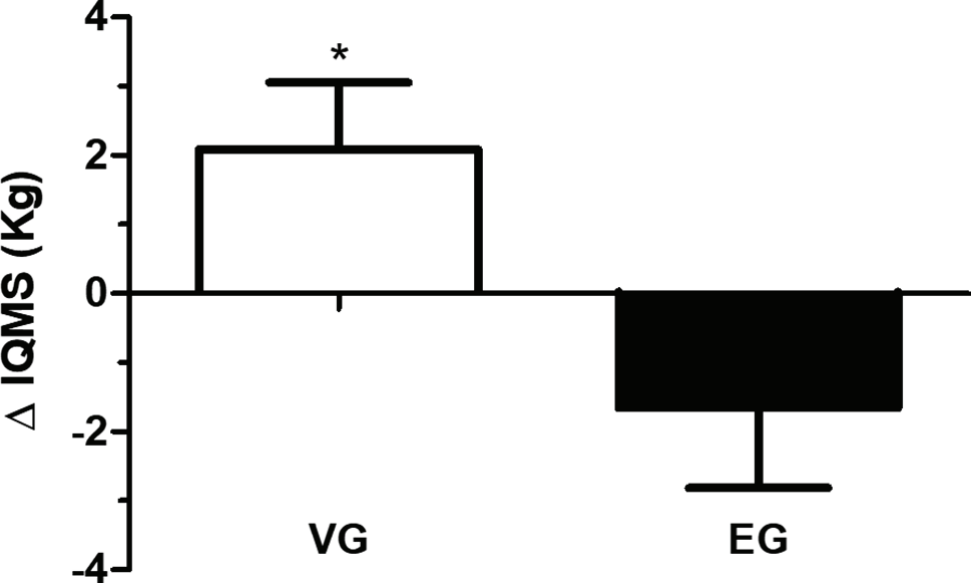
The delta (Δ) in isometric quadriceps muscle strength (IQMS) in the vibration group (VG) and exercise group (EG). The data are presented as the mean and standard error. ^*^VG is significantly different from EG (*p* ≤ 0.05). Data are presented in delta (Δ), i.e., the variation between the values measured before and after the intervention period (independent *t*-test).

The IQMS data, which were analyzed through the variation between the values measured before and after the intervention period, i.e., delta (Δ), showed a significant increase in the VG group compared to the EG group (IQMS; *p* ≤ 0.05; effect size = 1.3). After the intervention period, compared to the baseline of the study, the IQMS increased on average 2.17 kg (+13.48%) in the VG group and decreased on average 1.66 kg (−6.53%) in the EG group (Figure 2).

**Figure 3:**
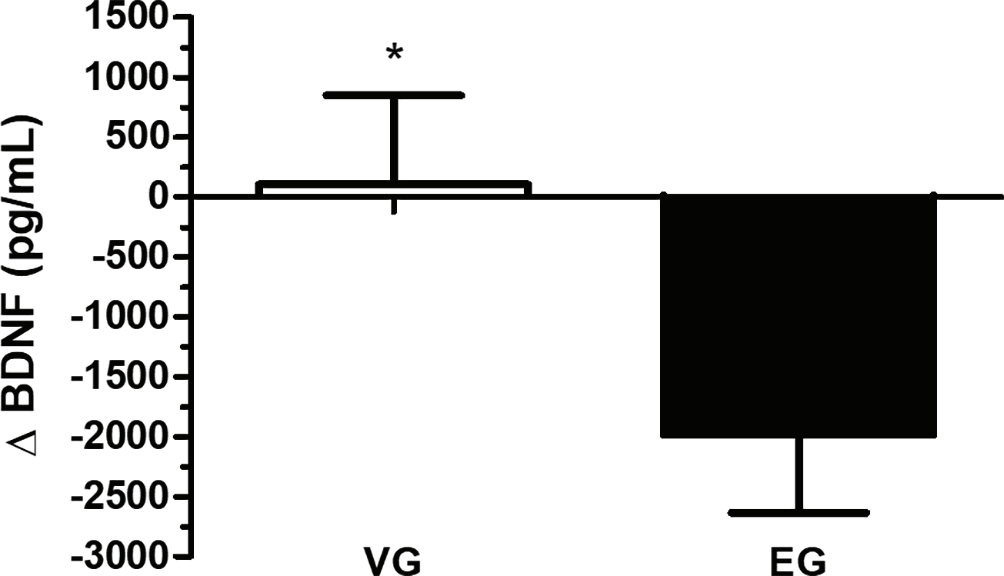
The delta (Δ) in brain-derived neurotrophic factor (BDNF) in the vibration group (VG) and exercise group (EG). The data are presented as the mean and standard error. ^*^VG is significantly different from EG (*p* ≤ 0.05). Data are presented in delta (Δ), i.e., the variation between the values measured before and after the intervention period (independent *t*-test).

**Figure 4:**
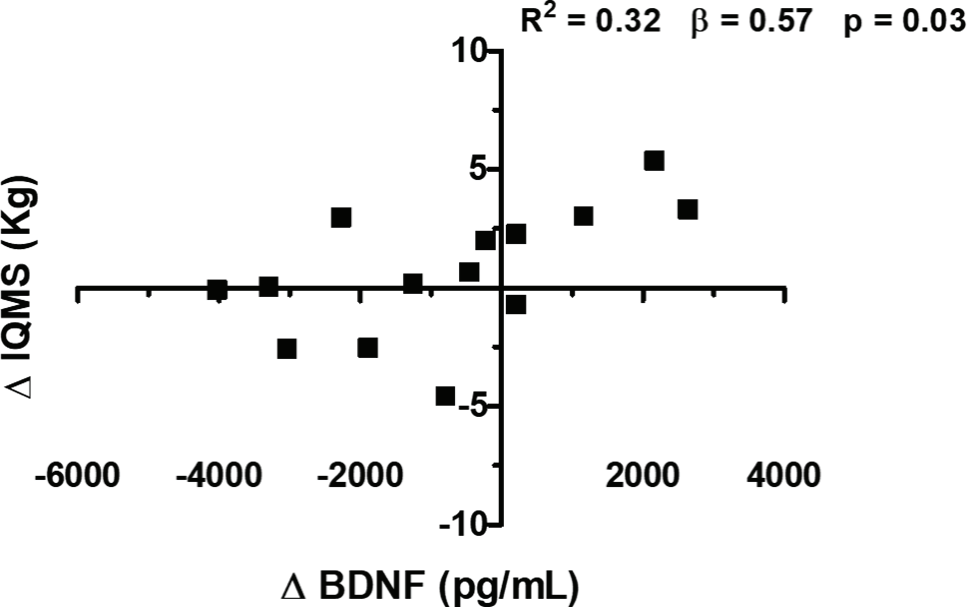
Correlation between the delta (Δ) of the isometric quadriceps muscle strength (IQMS) and plasma concentration of brain-derived neurotrophic factor (BDNF) in older women with osteoarthritis (OA) of the knee. β, standardized regression coefficient; *R*^2^, *R* square (Pearson correlation).

The Δ BDNF plasma levels demonstrated a significant increase in the VG group compared to the EG group (Δ BDNF; *p* ≤ 0.05; effect size = 1.1) (Figure 3). There was a positive correlation between BDNF plasma levels and IQMS (Figure 4). The linear regression analysis showed the prediction between Δ BDNF and Δ IQMS, where the increase in Δ BDNF seems to account for 56.9% of the Δ IQMS. Thus, for each increase of 1 pg/ml in the Δ BDNF, there will be an increase of 0.32 kg in Δ IQMS (standardized regression coefficient = 0.57; *R* square = 0.32; *p* = 0.03).

## Discussion

To our knowledge, this was the first randomized study designed to determine whether the addition of WBV to squat training could improve IQMS and affect the BDNF plasma in elderly women with kOA. The results of this study indicated that the addition of WBV to squat exercise training improved IQMS values and increased plasma BDNF concentrations in the studied population.

The increase in IQMS observed when adding WBV to squat exercise training in elderly people is consistent with previous literature (Roelants et al., 2004; Bogaerts et al., 2007; Rees et al., 2008). The chronic effect of WBV training in IQMS is in accordance with the hypothesis that the tonic vibration reflex primarily affects the subject’s ability to generate high firing rates in high-threshold motor units (Roelants et al., 2004). In addition, it has been suggested that the recruitment thresholds of the motor units during WBV is lower than with voluntary contractions, probably resulting in a more rapid activation and training of high-threshold motor units (Roelants et al., 2004).

However, it is important to mention that the studies cited above (Roelants et al., 2004; Bogaerts et al., 2007; Kean et al., 2010) were performed by elderly subjects with no history of joint damage. kOA appears to lead to a significant reduction in quadriceps muscle strength and, consequently, the ability to walk, climb stairs, and rise from a chair, especially in elderly people. Therefore, it is imperative to study the effect of the addition of WBV to squat exercise training in elderly people with kOA.

To our knowledge, there is only one study in the literature that investigated and demonstrated the effectiveness of WBV training on quadriceps muscle strength in individuals with kOA compared to a control group (without intervention; Trans et al., 2009). Although our results are in line with this prior study, some differences should be raised. In the study of Trans et al., the intervention group was subjected to solely WBV training, and the control group did not participate in any type of intervention. However, in the present study, the effects of adding WBV to squat training were evaluated. Moreover, in our study, there was a standardization of the knee flexion angle, and in the study of Trans et al., the participants flexed their knees until the position was self-perceived.

The increase in IQMS in the VG group has a significant clinical impact, as the literature indicates that clinically relevant minimum change is 2.25 Nm (Kean et al., 2010). Once 1 kg corresponds to 9.81 Nm, the increase of 2.17 kg in IQMS in the VG group corresponds to 21.29 Nm (a value close to the minimum clinically relevant). In EG group, there was a decrease in IQMS. The pain and the increase in intra-articular fluid, which are common in kOA, sensitize the capsular mechanoreceptors that send signals to medullary inhibitory interneurons. These interneurons inhibit alpha motor neurons and, consequently, reduce the signals that would be transmitted to the muscle groups, especially the quadriceps. This phenomenon, called arthrogenic muscle inhibition, is likely generated by the abnormal afferent information of the affected joint resulting in the decreased activation of the muscles that act there (McNair et al., 1996), progressing to a loss of muscle mass, decrease of muscle strength, and physical functioning (Sharma et al., 2003). Furthermore, age-related muscle loss is also a result of reductions in the size and number of muscle fibers, possibly due to a multifactorial process that involves physical inactivity, nutritional intake, oxidative stress, and hormonal changes corresponding to a condition known as sarcopenia. In addition, marked motoneuron loss and aberrant neuromuscular sprouting have been observed in aged mammals (Baumgartner et al., 1999). The reversal of this situation can be achieved by physical exercise that induces a cascade of molecular and cellular processes that support neuronal plasticity. However, the decrease in IQMS of the EG group demonstrated that the intensity of the exercise was not enough to preserve muscle mass.

The BDNF has been proposed as an essential neurotrophin related to neuronal plasticity (Knaepen et al., 2010; Teixeira et al., 2010) that plays an important role in old-age survival because of its role in preventing neuronal death during stress (Schabitz et al., 2007). The expression profiling of BDNF has shown that this neurotrophin is expressed differentially in skeletal muscle under various physiological and pathological conditions (Chevrel et al., 2006). Moreover, the expression of BDNF mRNA by human skeletal muscle increased after 2 h of cycle ergometer exercise (Matthews et al., 2009).

Therefore, we proposed in the present study to investigate the effect of adding WBV to squat exercise training on BDNF plasma concentrations. As a result, a significant increase in the VG group compared with the EG group was observed, supporting a possible neuromuscular adaptation hypothesis arising from this type of intervention in the studied population, as neurotrophins and neurotrophin receptors play a role in the coordination of muscle innervation and functional differentiation of neuromuscular junctions (Chevrel et al., 2006). This hypothesis is supported by the positive correlation found between plasma level of BDNF and IQMS.

Several studies have demonstrated that BDNF is a protein that is produced in skeletal muscle cells, and its expression is increased by contraction to enhance fat oxidation in an AMPK-dependent fashion, most likely by acting in an autocrine and/or paracrine manner within skeletal muscles (Bogaerts et al., 2007; Krabbe et al., 2009). Thus, an increase in BDNF plasma could play an important role in old-age survival because of its role in preventing neuronal death during stress (Schabitz et al., 2007). Thus, it seems plausible that the improvement in isometric quadriceps muscle strength in the vibration group could be related to an increase in BDNF level.

In the EG group, plasma BDNF levels showed a decrease after the intervention period. BDNF is a member of the neurotrophic factor family that plays key roles in regulating survival, growth, and maintenance of neurons in differentiation (Lindsay, 1994) and synaptic plasticity and synaptic transmission efficacy (Knaepen et al., 2010), and it is involved in muscle regeneration (Sakuma and Yamaguchi, 2011). Of the neurotrophins, BDNF, which is considered a contraction-inducible protein in skeletal muscle (Matthews et al., 2009), appears to be the most susceptible to regulation by exercise and physical activity (Knaepen et al., 2010; Coelho et al., 2012). However, it seems that performing only squatting exercises has not induced changes in BDNF plasma levels, preventing the degeneration of motoneurons and muscle fiber innervations in the elderly with knee OA. Inevitably, this study had some inherent limitations. First, the study was performed as a single-center trial with a relatively small number of participants. However, statistical analyses demonstrated a “large” effect size for IQMS (*p* ≤ 0.05; effect size = 1.1) and BDNF (*p* ≤ 0.05; effect size = 1.3). Moreover, considering the variability in the BDNF response to exercise and the influence of the BDNF polymorphism variant on such response, the analysis of only seven plasma BDNF samples should be interpreted with caution. Moreover, because specific frequency and amplitude were used, the findings of this study cannot be extrapolated to other parameters of vibration and cannot be generalized to the elderly population since the volunteers were female healthy and sedentary. Last, as we aimed to investigate the addition of WBV to squat exercise training, we have decided not to use a sound that mimicked the noise of the vibratory platform in the control group.

## Conclusion

The addition of WBV to squat exercise training improves lower limb muscle performance in elderly women with kOA, likely by increasing BDNF, suggestive of modulation in neuromuscular plasticity. Future studies should be designed to investigate the specific mechanism, including joint analyses.

## Data Availability

The datasets generated for this study are available on request to the corresponding author.

## Ethics Statement

This study was carried out in accordance with the recommendations of the ethical principles for research involving humans (Resolution 196-96 of the National Health Council of the Brazilian Ministry of Health) and Federal University of Jequitinhonha and Mucuri Valleys Ethics Committee. All subjects gave written informed consent in accordance with the Declaration of Helsinki. The protocol was approved by the Federal University of Jequitinhonha and Mucuri Valleys Ethics Committee (protocol no. 046/08) and was registered in ClinicalTrials.gov (protocol ID: NCT03918291).

## Author Contributions

The specific contributions of the authors are as follows: AS, VM, NA, and AL contributed to the conception and design of the study. AS, VM, NA, SF, RT-G, CN, CB, and AL contributed to analysis and interpretation of the data. AS, VM, NA, SF, RT-G, CN, CB, JS, AO, and AL contributed to drafting of the article. AS, VM, NA, SF, JS, AO, VR, HL, PF, MB-F, and AL involved in the critical revision of the article for important intellectual content. AS, VM, NA, SF, RT-G, CN, CB, JS, AO, VR, HL, PF, MB-F, and AL contributed to the final approval of the article. AS, NA, VR, HL, PF, and AL provided statistical expertise. AS, VM, NA, SF, RT-G, CN, CB, MB-F, and AL contributed to provision of study materials. AS, VM, NA, CB, MB-F, and AL contributed to administrative, technical, or logistic support.

### Conflict of Interest Statement

The authors declare that the research was conducted in the absence of any commercial or financial relationships that could be construed as a potential conflict of interest.
